# CSER: a gene regulatory network construction method based on causal strength and ensemble regression

**DOI:** 10.3389/fgene.2024.1481787

**Published:** 2024-09-20

**Authors:** Yujia Li, Yang Du, Mingmei Wang, Dongmei Ai

**Affiliations:** School of Mathematics and Physics, University of Science and Technology Beijing, Beijing, China

**Keywords:** causal strength, ensemble regression, gene regulatory network, key regulatory genes, colorectal cancer, biomarkers

## Abstract

**Introduction:**

Gene regulatory networks (GRNs) reveal the intricate interactions between and among genes, and understanding these interactions is essential for revealing the molecular mechanisms of cancer. However, existing algorithms for constructing GRNs may confuse regulatory relationships and complicate the determination of network directionality.

**Methods:**

We propose a new method to construct GRNs based on causal strength and ensemble regression (CSER) to overcome these issues. CSER uses conditional mutual inclusive information to quantify the causal associations between genes, eliminating indirect regulation and marginal genes. It considers linear and nonlinear features and uses ensemble regression to infer the direction and interaction (activation or regression) from regulatory to target genes.

**Results:**

Compared to traditional algorithms, CSER can construct directed networks and infer the type of regulation, thus demonstrating higher accuracy on simulated datasets. Here, using real gene expression data, we applied CSER to construct a colorectal cancer GRN and successfully identified several key regulatory genes closely related to colorectal cancer (CRC), including *ADAMDEC1*, *CLDN8*, and *GNA11*.

**Discussion:**

Importantly, by integrating immune cell and microbial data, we revealed the complex interactions between the CRC gene regulatory network and the tumor microenvironment, providing additional new biomarkers and therapeutic targets for the early diagnosis and prognosis of CRC

## 1 Introduction

Genes participate in cellular life activities through various pathways, including the encoding of proteins. For instance, genes can promote cell proliferation or inhibit apoptosis, thereby increasing the number of tumor cells (Dandoti, 2021). Consequently, genes have considerable impacts on the occurrence and development of cancer. It is essential to identify cancer-related genes because they typically regulate other genes and, in turn, affect cellular functions and behaviors, thereby stimulating the progression and deterioration of tumors (Douglas, 2022; Hanahan and Weinberg, 2011). Therefore, the study of genes and gene regulation has become an important topic in bioinformatics, and constructing GRNs has become an essential task. GRNs interconnect genes with different functions according to certain rules, transforming the relationships among genes into a highly complex network structure (Kaler et al., 2009). Gene regulation encompasses a spectrum of mechanisms involving transcription factors and other regulatory proteins encoded by regulatory genes that can either activate or repress gene transcription, thus controlling the expression levels of target genes and achieving intergenic regulation (Badia-I-Mompel et al., 2023). Key regulatory genes play a particularly significant role in GRN stability. The expression of key regulatory genes can affect cancer progression. For example, Zhang et al. (2023) analyzed the control hubs in a cancer gene regulatory network. By integrating experimental validation, they demonstrated that these genes are involved in multiple regulatory pathways and are associated with the proliferation of cancer cells (Zhang et al., 2023). Importantly, GRNs can help identify key regulatory genes related to cancer. The representative algorithms used to construct GRNs include algorithms based on correlation, such as weighted gene coexpression network analysis (WGCNA) (Zhang and Horvath, 2005), and parsimonious gene correlation network analysis (Care et al., 2019). Compared with other algorithms, algorithms based on correlation have certain advantages for constructing GRNs because of their reduced computational complexity. However, simple correlation could confuse direct and indirect regulatory relationships, leading to lower GRN accuracy. Additionally, algorithms based on conditional mutual information, such as conditional mutual inclusive information (CMI2) (Zhang et al., 2015), can distinguish between direct and indirect regulation but cannot determine the direction and type of regulation. Regression-based algorithms, such as TIGRESS (Adabor and Acquaah-Mensah, 2019), GENIE3 (Huynh-Thu et al., 2010), and PoLoBag (Ghosh et al., 2021), can infer the direction and type of regulation; however, their speed and accuracy may be limited by the sample features in the dataset. Furthermore, dynamic network inference algorithms based on temporal progression, such as PROB (Sun et al., 2021) and DryNetMC (Zhang et al., 2019), provide insights into the temporal dynamics of gene regulation.

To overcome the limitation noted above, we introduced a method of constructing GRNs based on causal strength and ensemble regression (CSER). WGCNA based on correlation has less complexity; thus, we initially employed it to efficiently select gene modules closely related to cancer based on their coexpression relationships. Since most GRNs are sparse (Kim et al., 2023), not all genes have regulatory relationships with each other. Therefore, we used another algorithm based on conditional mutual inclusive information focused on causal strength. This algorithm can quantify the correlation between genes, thereby removing indirect regulation and marginal genes, ensuring a stronger correlation between genes and improving network model accuracy. Finally, an ensemble regression algorithm was used to infer the direction and type of gene regulation, considering both linear and nonlinear features, to deduce the regulatory type—inhibition or activation—and construct the final directed GRN.

Genes can regulate immune cell activity and affect immune responses, and abnormal gene expression can affect cellular function, including immune cells. For example, mutant *p53* affects innate immunity and promotes cancer (Yang et al., 2014), whereas high *SOX17* expression in CRC reduces CD8^+^ T-cell infiltration, allowing cancer cells to evade immune surveillance (Zamarron and Chen, 2011). Immune cells infiltrate the tumor microenvironment, directly contacting tumor cells to promote (through tumor-promoting immune subsets, i.e., Tregs) or inhibit tumor cells growth, crucially influencing tumor occurrence and development (Shaul and Fridlender, 2017; Edin et al., 2019). Li et al. (2017) analyzed the types of immune cells in early-stage nonsquamous non-small cell lung cancer tissue in association with patient survival data and found higher neutrophil infiltration in high-risk groups, thus serving as an immune prognostic signature. Xiong et al. (2018) analyzed the proportion of tumor-infiltrating immune cells in colon cancer and found significant differences in immune infiltration characteristics between colorectal cancer tissue and adjacent tissue. These studies indicate subtle differences in the composition of immune cells infiltrating the normal microenvironment and the colorectal cancer (CRC) microenvironment, which may, in turn, be important determinants for cancer recognition and therapeutic response. Therefore, we considered both gene regulation and immune cell deployment by combining key regulatory genes and differential immune cell ratios as features and applying a support vector machine (SVM) algorithm to classify samples, thereby improving the accuracy of our cancer recognition classifier.

With the continuous improvement in the depth of our understanding of the tumor microenvironment (TME), increasing evidence has indicated the existence of intratumor microbiomes in mucosal-site cancers, such as lung, colorectal, and esophageal cancers (Azevedo et al., 2020; Wong-Rolle et al., 2021; Cogdill et al., 2018). In addition, since fungi and other microbes in tumor tissues may play complex roles in cancer development, microbiota is a potentially important component of TME (Xie et al., 2022). Triner et al. (2019) reported that microbes in tumors induce the production of IL-17, promoting B-cell entry and tumor growth, while neutrophils can limit the tumor microbiome. In several types of cancers, especially gastrointestinal cancers, the microbiome is an important cause of DNA damage. DNA damage can lead to an increase in genetic mutations and ultimately may lead to tumors. Thus, genes, immune cells, and microbes all interact, affecting tumor development, which we have considered in our integrated GRN approach. Compared with normal colon tissue, CRC tissue is rich in *Fusobacterium*, which is negatively correlated with recurrence-free survival, indicating poor prognosis (Kostic et al., 2012; Yu et al., 2017). Thus, microbe-based detection may serve as a noninvasive diagnostic or prognostic tool for colorectal cancer screening.

**FIGURE 1:**
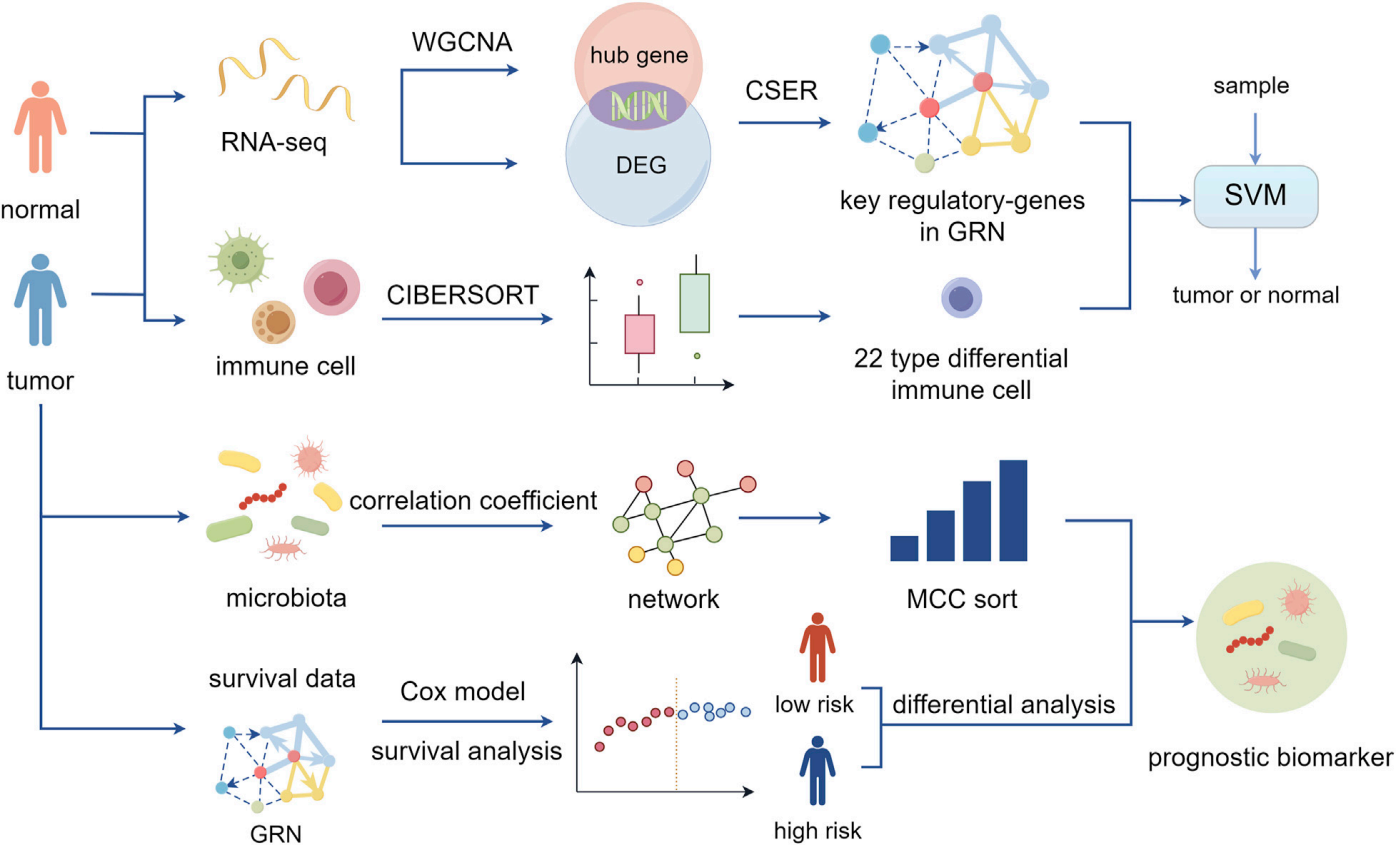
Schematic of this study. Using CSER to construct the gene regulatory network and identify key regulatory genes of colorectal cancer. Combining differential immune cell ratios to achieve diagnostic classification of patient samples. Performing prognostic risk assessment and microbial signature analysis (By Figdraw).

Considering the aforementioned findings, we constructed a risk model based on key genes in the CRC GRN, combining gene expression and survival data to calculate a risk score that closely aligns with colorectal cancer patient prognosis. Then, based on the risk score, tumor samples were divided into high-risk and low-risk groups for differential analysis to obtain differential microbes that yielded microbial characteristics related to prognosis when combined with the microbial interaction network. The overall analysis process of this study is shown in Figure 1. CRC-related biomarkers from gene expression, the immune cell ratio, and microbial abundance were determined.

## 2 Materials and methods

### 2.1 Datasets

We used gene expression profile data from The Cancer Genome Atlas (TCGA), including 44 normal and 571 CRC samples. Preprocessing steps were employed to ensure the uniqueness of the gene expression levels. After calculating the mean values of duplicated gene expression and removing low-expression mRNAs, a total of 14,325 gene expression profiles were obtained from 615 samples. Clinical data for 548 colorectal cancer patients were also downloaded, including patient IDs, survival times, and survival statuses. By merging clinical data with gene expression profiles, 473 colorectal cancer samples were finally obtained with clinical and gene expression profile data.

Three simulated datasets were used to evaluate the performance of CSER, all possessing standard networks. The simulated datasets were downloaded from the DREAM4 challenge, which provides gene expression data for yeast and the corresponding standard networks (Schaffter et al., 2011). Supplementary Table 1 shows detailed information on the datasets.

Microbial relative abundance data, including the relative abundance of 2,852 microbes in 153 colorectal cancer samples, were obtained from Ai et al. (2023). To ensure the validity of subsequent statistical analyses, we selected microbes present in at least 80% of the samples and ensured that each sample contained at least 80% of the microbial abundance data. After screening, the relative abundance of 15 microbes in 143 CRC samples was obtained.

### 2.2 Gene regulatory network construction algorithm

CSER quantifies causal gene relationships, alleviates the overestimation of mutual information and the underestimation of conditional mutual information, and improves the accuracy of the regulatory network. First, WGCNA clustered all genes into modules to identify cancer-related hub genes. Subsequently, the causal strength between genes was calculated using conditional mutual inclusive information, and independent genes, i.e., genes with no relationship, were removed to form the initial network. Finally, a GRN was constructed using the remaining genes based on an ensemble regression algorithm, resulting in a GRN with both directionality and regulatory type, reflecting activation and repression effects on the target gene.

#### 2.2.1 Weighted gene coexpression network analysis

WGCNA (Zhang and Horvath, 2005) is commonly used to study the correlation between phenotypic traits and genes because it can cluster genes with similar expression patterns into modules. Through WGCNA, it is possible to identify gene modules with similar expression within a large number of genes and determine the association between the modules and the phenotype of interest. WGCNA assumes that gene networks follow a scale-free distribution, and most real biological networks are scale-free networks (Barabasi and Bonabeau, 2003). Specifically, in a scale-free network, a small number of nodes exhibit a degree much higher than the average degree. These nodes are referred to as hub nodes and are connected to many other nodes; thus, they play a dominant role in scale-free networks.

To establish a weighted gene coexpression network involves studying the mutual relationship between two genes. The similarity $s_{ij}$ between gene i and gene j is represented in Equation 1:

$$s_{ij}=\left|cor\left(x_{i},x_{j}\right)\right|$$ (1)

where the gene expression matrix G can be transformed into a similarity matrix $S=s_{ij}$ . WGCNA employs a soft threshold approach to calculate the correlation between genes. The correlation between any two genes i and gene j is measured by the adjacency coefficient $a_{ij}$ , which is computed as shown in Equation 2:

$$a_{ij}={\left|s_{ij}\right|}^{\beta }$$ (2)

where the exponent β represents the soft threshold. Applying the power function to gene correlation coefficients minimally affects strong correlations, whereas weaker correlations exhibit a significant decrease. Raising the correlation coefficients to the power of β weakens already weak correlations, transforming the gene connectivity network into a scale-free network. After eliminating weak correlations and retaining those with biological significance, hierarchical clustering is conducted based on the dissimilarity between genes to obtain gene modules, which are subsequently screened. Ultimately, hub genes are identified based on gene and module significance.

#### 2.2.2 Quantifying gene associations based on causal strength

CMI2 (Zhang et al., 2015) is an effective unbiased measurement method based on causal strength (Janzing et al., 2013) that can quantify causal relationships between genes. In other words, in a directed acyclic graph, if gene B is directly regulated by gene A or indirectly regulated through gene C, the association between A and B is defined in Equation 3:

$$CMI2\left(A,B|C\right)=\left(D_{KL}\left(P\|P_{A\to B}\right)+D_{KL}\left(P\|P_{B\to A}\right)\right)/2$$ (3)

where $P=P\left(A,B,C\right)$ is the joint probability distribution of A,B, and C , $P_{A\to B}=P_{A\to B}\left(A,B,C\right)$ is the intervention probability distribution after removing edge A→B , and similarly, $P_{B\to A}=P_{B\to A}\left(A,B,C\right).\;D_{KL}\left(P\|P_{A\to B}\right)$ is the Kullback‒Leibler (K–L) divergence from P to $P_{A\to B}$ ; similarly, for $D_{KL}\left(P\|P_{B\to A}\right)$ . CMI2 has an order $\left|C\right|$ , representing the number of conditional genes C , and mutual information is the zero-order CMI2.

The probability $P_{A\to B}$ is defined in Equation 4:

$$P_{A\to B}\left(a,b,c\right)=P\left(a,c\right)\sum_{a}P\left(b|c,a\right)P\left(a\right)$$ (4)

where $P\left(b|c,a\right)$ is the conditional probability. According to the definition of K-L divergence. The definition of $D_{KL}\left(P\|P_{A\to B}\right)$ is given in Equation 5:

$$D_{KL}\left(P\|P_{A\to B}\right)=\sum_{a,b,c}P\left(a,b,c\right)\ln\frac{P\left(a,b,c\right)}{P\left(a,c\right)\sum_{a}P\left(b|c,a\right)P\left(a\right)}$$ (5)

CMI2 can be decomposed as shown in Equation 6:

$$CMI2\left(A;B|C\right)=CMI\left(A;B|C\right)+\frac{1}{2}D_{KL}(P\left(B\left|C)\|P_{A\to B}\left(B|C\right)+\frac{1}{2}D_{KL}(P(A\right|C\right)\|P_{B\to A}\left(A|C\right)$$ (6)

where $CMI\left(A;B|C\right)$ is conditional mutual information. If the second and third terms are 0, meaning that A and B are independent of C, then CMI2 is equal to CMI. Since the K–L divergence is nonnegative, the CMI2 between A and B given C is not less than the conditional mutual information between A and B given C.

Assume a gene expression matrix $G\in R^{n\times m}$ , where n represents the number of genes and *m* represents the number of samples. First, a complete connected graph is generated based on the number of genes. Second, for adjacent gene pairs *i* and *j*, calculate their mutual information. If gene pair *i* and *j* have low mutual information, remove the edge between genes *i* and *j*. Finally, for adjacent gene pairs *i* and, calculate the first-order CMI2 given another neighboring gene *z*. If the gene pair *i* and *j* have a low CMI2, remove the edge between them. This method can eliminate indirect regulation between genes while determining causal relationships.

Since most GRNs are sparse, some genes in the network may not have regulatory relationships with all other genes, and some regulatory relationships may be weak. Therefore, in this study, genes were selected by calculating CMI2 and removing independent genes from the network. Subsequently, the remaining genes were used to construct a regulatory network based on an ensemble regression algorithm.

#### 2.2.3 Regulatory inference based on ensemble regression algorithm

PoloBag (Ghosh et al., 2021) is an ensemble regression algorithm that divides the regulatory network construction problem into separate regression tasks for each target gene. Each regression task is performed using an ensemble of Lasso models within the bagging framework (Wang et al., 2011) trained on bootstrap samples. The bootstrap sample includes polynomial features, encompassing both linear features, i.e., randomly selected gene characteristics, and nonlinear features, i.e., those obtained by multiplying gene characteristics. Averaging the Lasso coefficients estimated from each bootstrap sample produces corresponding weights that can be positive or negative. Gene expression data comprise the input data for this algorithm, such that $D\in R^{n\times m}$ represents the input gene expression data for n genes across m samples. In this study, the input gene expression data consisted of 174 genes and 615 samples. For $n_{R}$ potential regulatory genes in the network, the purpose of constructing the network is to identify the positive and negative edge weight vectors $w\in R^{n_{R}\left(n-1\right)\times 1}$ between regulatory genes and target genes. These weights represent the strength and type (activation/repression) of regulatory interactions. In the absence of prior knowledge of regulatory genes, all genes are considered potential regulatory genes, $n_{R\;}$ *= n*.

PoLoBag can determine the regulatory relationships between regulatory genes and target genes, including the direction of regulation and the type of regulatory effect, whether activation or repression.

### 2.3 Immune cell proportion algorithm

CIBERSORT (Newman et al., 2015) is a computational method based on linear support vector regression that can estimate the proportion of immune cells from gene expression profile data. CIBERSORT is particularly useful for analyzing immune cell infiltration in the tumor immune microenvironment and calculating the relative abundance of different immune cells in tumor tissues.

By integrating feature selection and robust mathematical optimization techniques, CIBERSORT effectively amplifies the performance of deconvolution analysis. For feature matrices composed exclusively of immune cell types, it is possible to filter out nonhematopoietic and cancer-specific genes to mitigate the impact of nonimmune cells on the deconvolution results. Additionally, CIBERSORT improves the stability of the signature matrix and further reduces the effects of multicollinearity by incorporating a function that minimizes the condition number.

### 2.4 Support vector machine algorithm

SVM is a commonly used binary classification method employed with the fundamental idea of finding the optimal hyperplane in multidimensional space. The SVM algorithm can simplify complex classification and regression tasks when handling small samples, thereby improving the efficiency and accuracy of the algorithm. The SVM algorithm, known for its streamlined structure, robust generalization capabilities, and minimal parameter requirements, has broad application across various fields. By employing kernel functions, SVM overcomes dimensionality disasters and nonlinear separability, thus avoiding increased computational complexity.

### 2.5 Cox proportional hazards model

The Cox proportional hazards model (Cox model) (Samar et al., 2021) is commonly used to explore whether genes affect patient survival through survival analysis models. The model can analyze the impact of multiple genes on survival time and identify factors that pose significant risks to patients.

The Cox model is given in Equation 7:

$$h\left(t\right)=h_{0}\left(t\right)\exp\left(\alpha_{1}Y_{1}+\alpha_{2}Y_{2}+\cdots +\alpha_{p}Y_{p}\right)$$ (7)

where $Y_{1},Y_{2},\cdots ,Y_{P}$ are variables that might affect survival, such as gene expression levels; $h\left(t\right)$ is the hazard function at time t ; $h_{0}\left(t\right)$ is the baseline hazard function, where the independent variables are all set to 0; and $\alpha_{1},\alpha_{2},\cdots ,\alpha_{P}$ are the partial regression coefficients of the variables, which can be estimated from the data. In the Cox model, if the partial regression coefficient $\alpha_{i}$ is greater than 0, the corresponding variable is considered a high-risk factor; if it is less than 0, it is considered a protective factor.

## 3 Results and discussion

### 3.1 Performance evaluation

To evaluate the performance of our algorithm, we defined its accuracy in Equation 8:

$$Accuracy=\frac{TP+TN}{TP+TN+FP+FN}$$ (8)

where TP and TN represent the number of activations and repression correctly inferred, respectively. FP represents the number of inhibitions incorrectly inferred as activations. FN represents the number of activations incorrectly predicted as inhibitions.

We conducted benchmark tests on three simulated datasets, which possess standard networks with directed and signed regulations. For each dataset, the initial network of gene interactions was obtained by first calculating the CMI2 values based on causal strength. We then conducted a gene selection process by removing isolated genes from the initial network. By leveraging the expression data of these screened genes, the GRN was constructed by quantifying the regulatory interactions utilizing the ensemble regression algorithm. The accuracy of the three simulated datasets is shown in Table 1, and the results indicate that CSER outperforms PoLoBag.

**TABLE 1:** Accuracy on simulated datasets.

Algorithm	Dataset A	Dataset B	Dataset C
PoLoBag	0.73	0.71	0.70
CSER	0.75	0.78	0.72

### 3.2 Colorectal cancer gene regulatory network construction

Constructing the gene regulatory network on real gene expression profile data depends on gene selection. Therefore, WGCNA was initially employed to identify cancer-associated hub genes within the coexpression network. Since cancer occurrence is typically related to abnormal gene expression, the Wilcoxon test was employed to identify differentially expressed genes (DEGs) between normal and tumor samples. Then, considering both coexpression and differential expression characteristics, we intersected the hub genes with the DEGs and verified that all hub genes showed expression differences between the two sample types. We further identified the key regulatory genes in the network, ultimately identifying biomarkers associated with CRC.

#### 3.2.1 Hub genes related to colorectal cancer

Based on CRC gene expression data downloaded from the TCGA database, WGCNA was used to identify CRC-related hub genes. First, the soft threshold β = 14 was determined to establish a scale-free network, followed by hierarchical clustering and differentiation using various colors.

**FIGURE 2:**
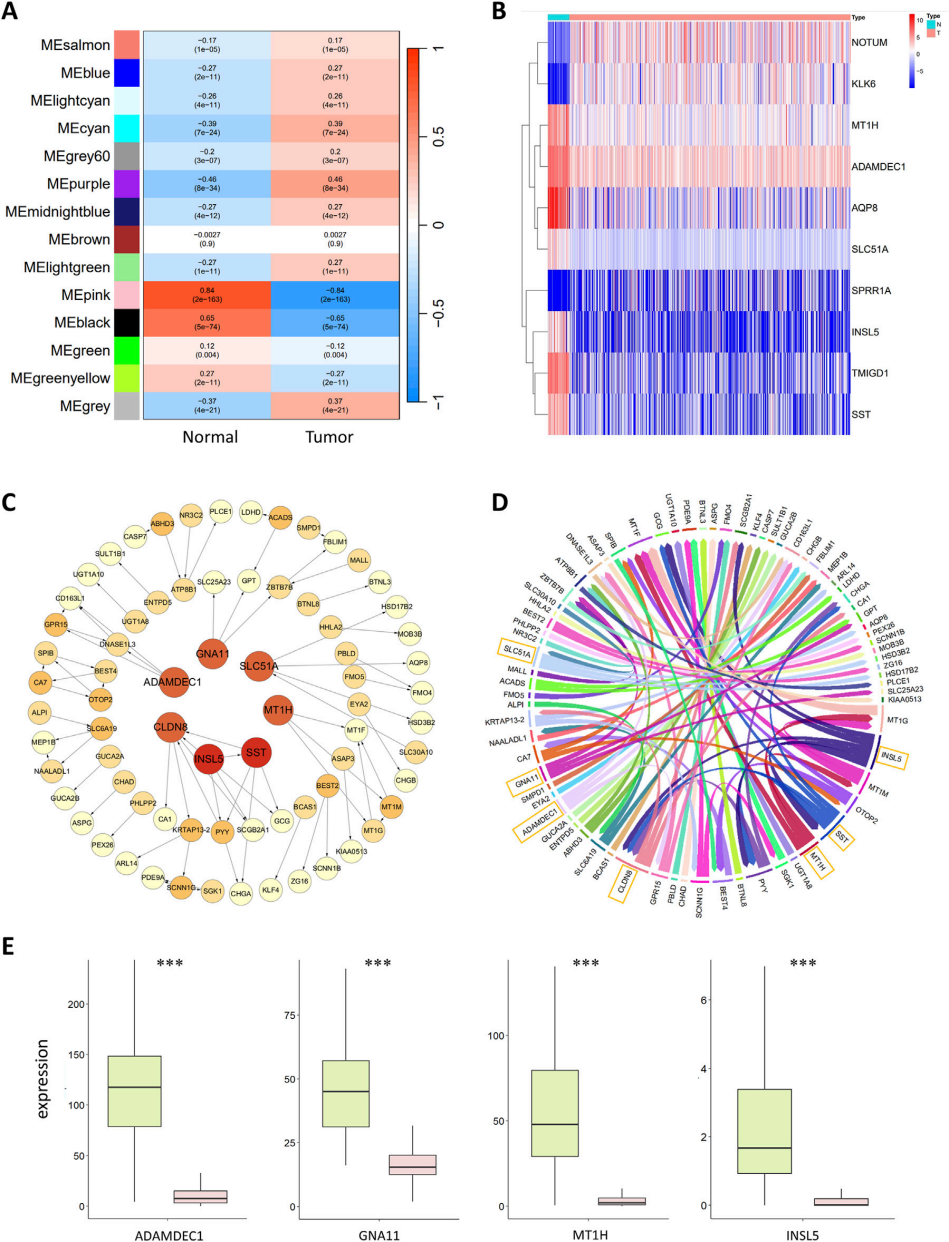
**(A)** Correlation analysis of gene coexpression modules with clinical phenotypes. Each row represents a unique gene coexpression module. The values enclosed in parentheses are the p values, with the numerical values outside indicating the correlation coefficients. Red denotes a positive correlation, while blue indicates a negative correlation. **(B)** Heatmap of differentially expressed genes. The horizontal axis represents the samples, with blue representing normal samples and pink representing tumor samples. The vertical axis represents the genes. The colors in the heatmap represent the expression levels of genes in the samples, with red indicating high expression and blue indicating low expression. **(C)** Colorectal cancer gene regulatory network. The circles in the graph represent genes, and the lines between them represent regulatory relationships. The tail of the arrow connects the regulatory gene, and the head connects the target gene, with the arrow indicating an activative relationship. Light yellow represents the target genes; the deeper the color of the gene is, the greater the out-degree is, indicating that the gene has more regulatory relationships. The red circles in the central area represent the seven most critical regulatory genes. **(D)** Chord diagram of the colorectal cancer gene regulatory network. Each color represents a gene, and the arrows point to the target genes. The genes with greater regulatory relationships correspond to a greater width. The seven most critical regulatory genes with the widest lines are marked in the diagram. **(E)** Boxplot of key regulatory genes, with green representing normal samples and red representing tumor samples. The central line within each box represents the median of the dataset.

During the construction of the WGCNA coexpression module, close connections with tumors were established to identify genes closely related to CRC. The correlations between the gene modules and the normal and tumor sample groups were calculated to identify modules closely related to tumors. The results are shown in Figure 2A. The MEpink module had the highest Pearson correlation coefficient with normal samples, with a value of 0.84 and *p* < 0.01, indicating a significant correlation. This finding suggested that MEpink is a key module closely related to tumors and that the genes in this module are associated with the occurrence and development of CRC.

MEpink contains 235 genes, some of which were identified as hub genes. The conditions for screening hub genes were GS > 0.5 and MM > 0.5, resulting in 174 hub genes. GS refers to the correlation of the gene with normal or tumor samples; the larger the GS is, the greater the correlation of the gene with normal or tumor samples will be. MM represents the correlation of a gene with the coexpression module; the larger the MM is, the more important the gene is.

#### 3.2.2 Differential gene expression analysis

In this study, the limma package (Ritchie et al., 2015) in R was used to analyse the preprocessed gene expression profile data. Gene expression data were divided into a healthy group (44 samples) and a tumor group (571 samples) for differential expression analysis. The Wilcoxon test was used for gene screening to identify DEGs between normal and tumor samples, and the p values were adjusted using the FDR correction package in R language. The conditions for screening DEGs were as follows: $\left|\log FC\right|>1$ , i.e., genes with more than twofold differences in expression between healthy individuals and cancer patients and a corrected p-value less than 0.05. The formula for calculating the log⁡FC is illustrated in Equation 9:

$$logFC=\log_{2}fold\;change=\log_{2}\frac{mean\;for\;tumor\;groups}{mean\;for\;normal\;groups}$$ (9)

Based on the above conditions, 3,676 DEGs were obtained, including 2,216 upregulated and 1,460 downregulated genes. A heatmap of the 10 significantly upregulated and downregulated DEGs is shown in Figure 2B.

Studies show that high expression of *SPRR1A* is associated with lymph node metastasis and low survival rates in CRC patients, and *SPRR1A* may serve as a potential prognostic biomarker for CRC (Deng et al., 2020). *AQP8* inhibits the growth and metastasis of colorectal cancer cells by downregulating PI3K/AKT signaling and reducing the expression of *PCDH7* (Wu et al., 2018). *TMIGD1* is a highly downregulated gene in CRC, and overexpression of the *TMIGD1* protein significantly impairs the metastatic and proliferative capacity of CRC cells. In contrast, the downregulation of *TMIGD1* may promote CRC progression; therefore, *TMIGD1* may serve as a prognostic biomarker for CRC (Mu et al., 2022). *NOTUM* is associated with the proliferation and migration of CRC cells, and *NOTUM* also has potential as a biomarker and therapeutic target for colorectal cancer (Yoon et al., 2018). *KLK6* expression is significantly upregulated in the tissues and serum of colorectal cancer patients and is closely related to poor prognosis; thus, *KLK6* may also be a potential CRC biomarker and therapeutic target (Kim et al., 2011).

#### 3.2.3 Colorectal cancer gene regulatory network based on the CSER

Following WGCNA and differential analyses, 174 hub genes and 3,676 DEGs were identified, with 174 intersecting genes between these two sets. The CMI2 value between genes was calculated using the gene expression profile data of these 174 intersecting genes in 615 samples, setting the threshold at 0.03. Genes with CMI2 values less than 0.03 were considered unrelated and formed the initial network. Independent genes were removed from this network; thus, the initial network contained no independent genes. Subsequently, PoLoBag was used to analyze the gene expression profile data of the 174 genes, with a focus on regulatory relationships with absolute weights greater than 0.5. This process revealed 71 regulatory relationships involving 74 genes. The CRC GRN was visualized using Cytoscape (Shannon et al., 2003) and R, as illustrated in Figures 2C, D.

The top 7 genes in the GRN, ranked by their out-degree, were identified as key regulators of CRC: *ADAMDEC1*, *CLDN8*, *GNA11*, *INSL5*, *MT1H*, *SLC51A*, and *SST*. These genes were used as features to discriminate between normal and tumor samples. Their expression shows significant differences between normal and tumor samples (with FDR-corrected *p* < 2e-18), and Figure 2E displays the data distribution in the two types of samples. Previous studies have shown that *ADAMDEC1* expression is lower in adenomatous and CRC tissues than in normal colorectal tissue, suggesting its involvement in colorectal adenoma development (Galamb et al., 2008). Compared to that in normal tissues, the protein expression of *CLDN8* is greater in colorectal cancer tissues, promoting the growth of CRC cells. *CLDN8* increases the proliferation, migration, and invasion of CRC cells by activating the MAPK/ERK signaling pathway, exhibiting an oncogenic effect on the progression of human CRC (Cheng et al., 2019). Decreased *GNA11* expression is a characteristic of advanced CRC, with mutations in *GNA11* disrupting the MAPK signaling pathway and enabling unchecked cell proliferation (Ziolko et al., 2015). The *MT1H* gene, part of the *MT1* subtype of metallothionein genes, has demonstrated tumor suppressor activity and downregulated expression in CRC (Han et al., 2013). Mashima et al. (2013) previously reported that *INSL5* might be a unique marker for the colorectum. Yang et al. (2021) reported that *INSL5* is more highly expressed in normal tissues than in tumor tissues and that the overexpression of *INSL5* significantly inhibits the proliferation of CRC cells, which is correlated with a better prognosis. *SST* and its analogs negatively regulate cancer growth, invasion, and metastasis by binding to specific receptors on tumor cells (Pyronnet et al., 2008). For example, the cytotoxic *SST* analog AN-162 inhibits human colon cell growth by inducing cell cycle arrest (Hohla et al., 2010).

The above studies indicate that the abnormal expression of key regulatory genes in the network is closely associated with the development of CRC. These genes promote or inhibit CRC progression through various biological mechanisms, including directing protein synthesis, regulating signaling pathways, and affecting cell proliferation. The identification of these key regulatory genes demonstrates the outstanding identification capability of CSER and provides potential therapeutic targets for CRC. Future studies should further integrate biological experiments to explore and validate the interactions among these genes.

### 3.3 Biomarkers for colorectal cancer diagnosis

**FIGURE 3:**
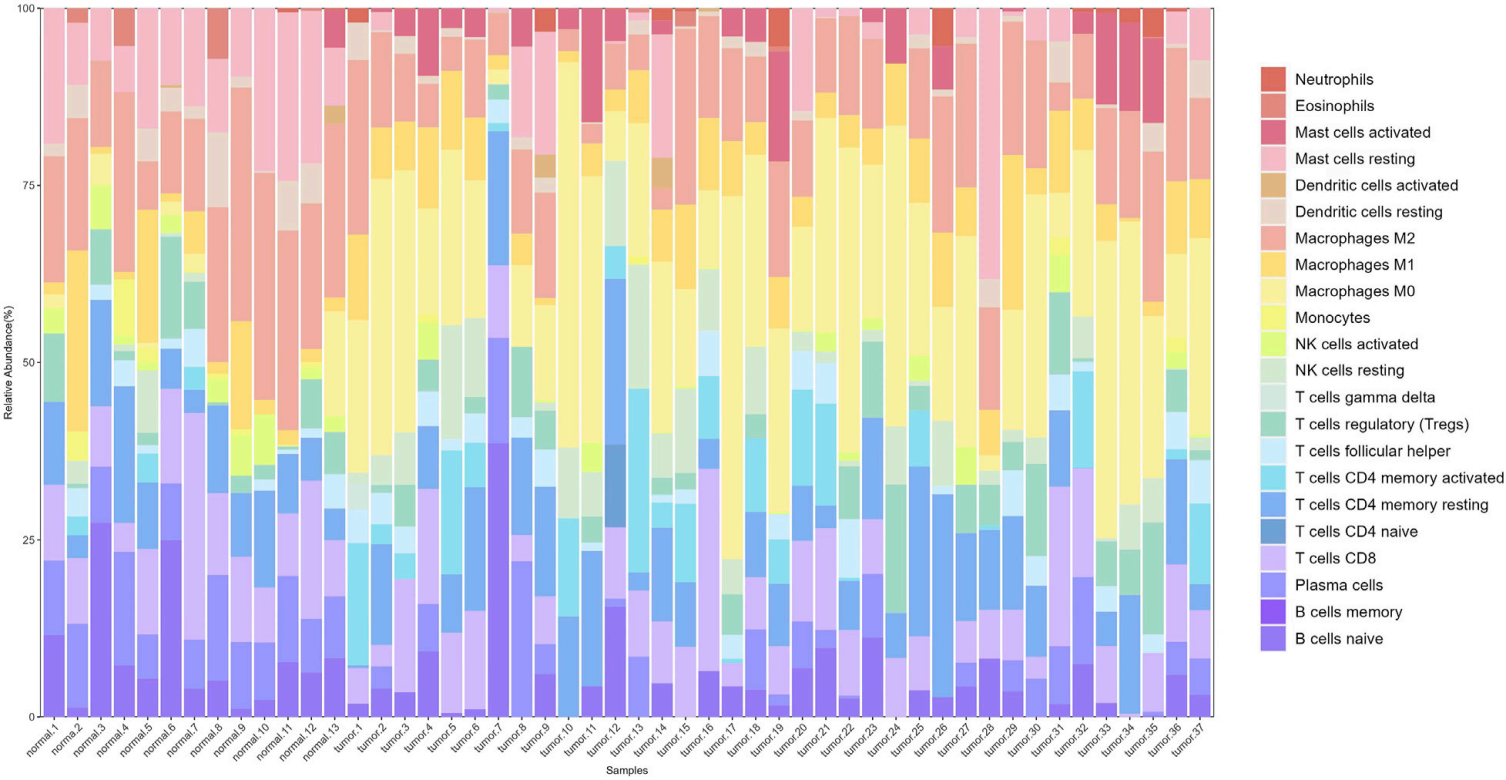
Stacked bar plot of immune cell proportions. The *x*-axis represents 50 samples, including 13 normal and 37 tumor samples. The *y*-axis represents the percentage of immune cells.

After preprocessing the gene expression profile data, including removing genes with low expression and performing normalization, we used the CIBERSORT algorithm to estimate the relative proportions of 22 types of immune cells in the samples. Filtering the results with *p* < 0.05 yielded immune cell proportions for 277 samples (13 normal and 264 tumor samples). A stacked plot of the relative proportions of 22 types of immune cells in some samples is shown in Figure 3.

Significant differences in immune cell composition between colorectal cancer and normal intestinal tissues were observed. Specifically, tumor tissues exhibited higher infiltration levels of activated mast cells and M0 macrophages. As early and abundant infiltrators in the TME, macrophages play a critical role in tumor progression. They are classified into M0, M1, and M2 subtypes based on their activation status, each with distinct immune functions.

**FIGURE 4:**
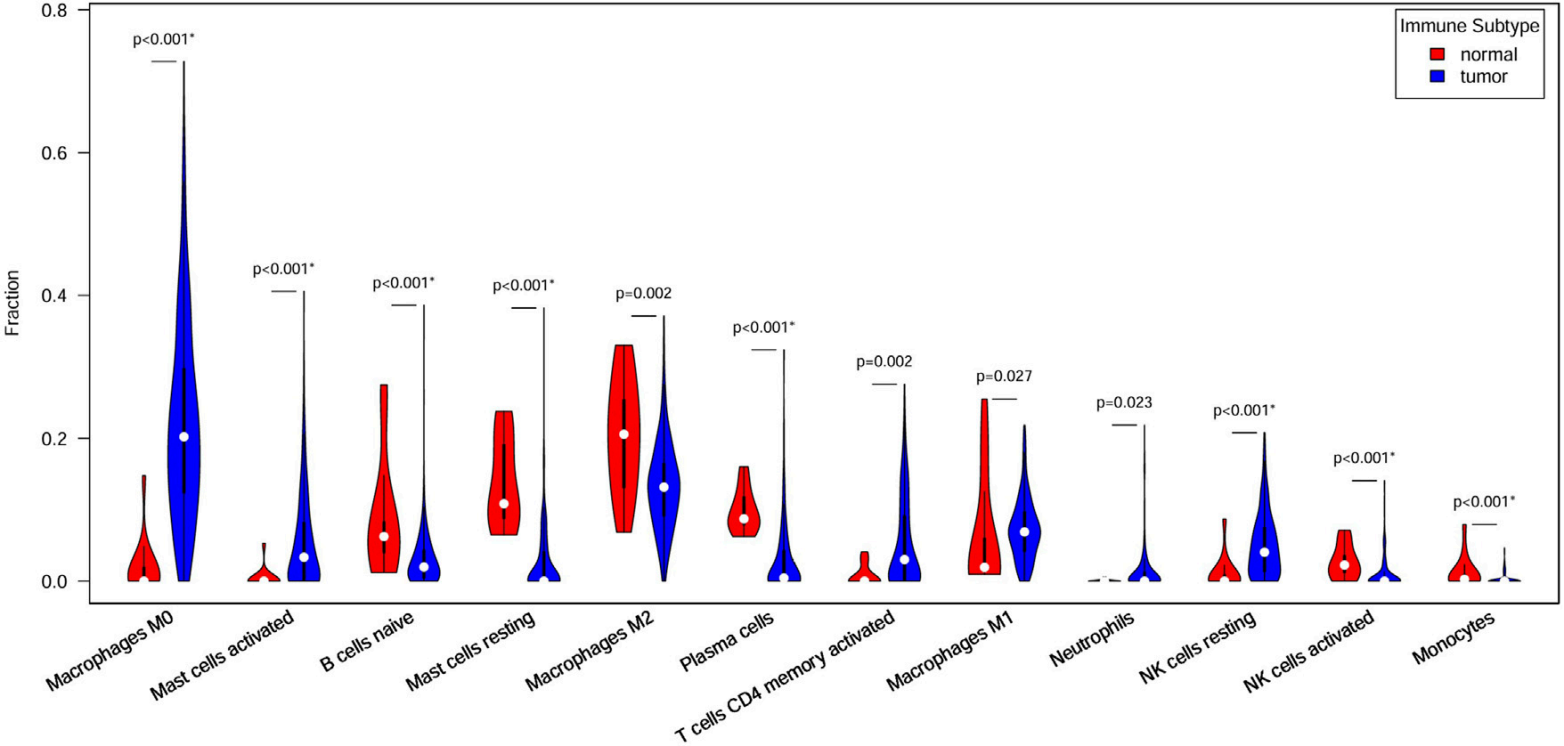
Immune cells with different ratios between normal and tumor samples. Red indicates normal samples, blue indicates tumor samples, and white dots denote median proportions.

Figure 4 shows 12 immune cell types with significantly differential proportions between normal and tumor samples. The findings of previous studies support our findings. For example Stanilov et al. (2014), reported that monocytes from advanced cancer patients secrete more TNF-α than monocytes from early-stage patients. TNF-α is closely linked to tumor promotion and progression; thus, the infiltration of monocytes is closely associated with CRC survival risk. Studies have reported that macrophages and IL-1 enhance Wnt signaling, thereby increasing transcriptional activity and promoting the growth of colon cancer cells (Kaler et al., 2009; Gao et al., 2017). Wu et al. (2020) reported that increased monocyte and macrophage infiltration is correlated with poor CRC patient prognosis.

The 7 key regulatory genes and the 12 significantly different immune cells were used as input features for an SVM classifier. The AUC increased from 0.77 to 0.99 when gene and immune cell features were combined, indicating strong classification performance. Thus, these 7 key genes and 12 types of immune cells can be considered biomarkers for predicting CRC.

### 3.4 Microbial signature and risk score calculations for prognosis

**FIGURE 5:**
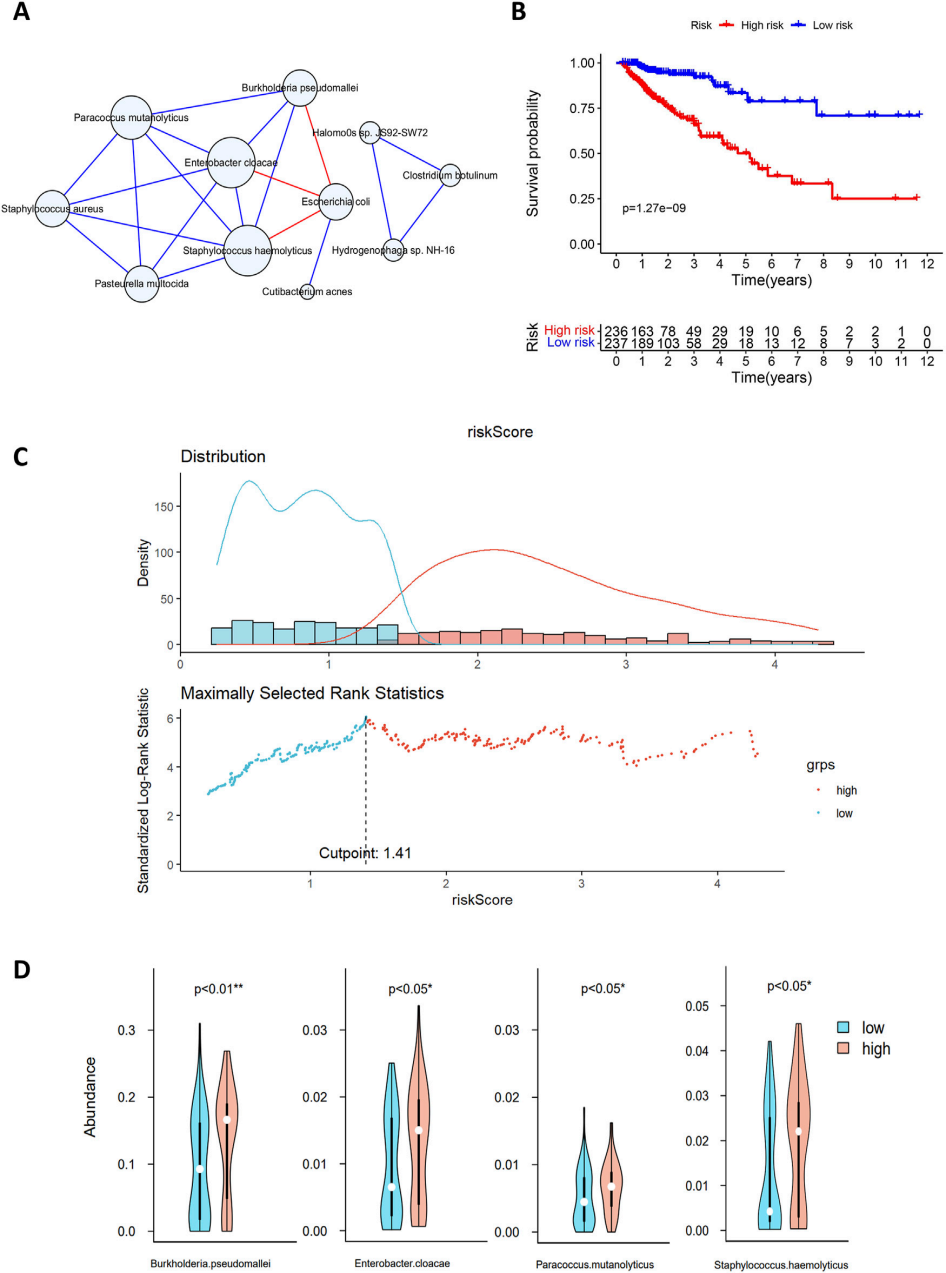
**(A)** Microbial interaction network. The blue circles represent microorganisms, and the lines between nodes indicate interactions, with blue and red lines indicating positive and negative correlations, respectively. **(B)** Survival analysis of the high-risk and low-risk groups based on gene risk scores. The *x*-axis represents survival time in years, and the *y*-axis represents survival rate. The numbers indicate the number of patients remaining at each time point. **(C)** Multivariate Cox analysis risk assessment grouping. The blue dots and red dots represent the low-risk group and high-risk group, respectively. **(D)** Differences in the abundances of microorganisms between the two groups. Blue indicates high-risk samples, red indicates low-risk samples, and white dots denote median abundance.

Spearman correlation coefficients were calculated based on the relative abundance of 15 microbial types in 143 CRC samples, resulting in a microbial abundance correlation matrix. Excluding low and nonsignificant correlations (correlation coefficient <0.7 and *p* > 0.05), we ultimately identified 11 interactions between microorganisms. The interactions were visualized using Cytoscape, and the resulting interaction map is depicted in Figure 5A. CytoHubba (Chin et al., 2014) provides a variety of analytic algorithms for assessing the importance of nodes. Key microorganisms in the network were ranked by their Matthews correlation coefficient (MCC) so that the top-ranked microorganisms were considered the key microorganisms in the interaction network. For a given microbial node v , the MCC of v is defined as shown in Equation 10:

$$MCC\left(v\right)=\sum_{C\in S\left(v\right)}\left(\left|C\right|-1\right)!$$ (10)

where $S\left(v\right)$ represents the set of the largest community that includes node v , and $\left(\left|C\right|-1\right)!$ denotes the product of all positive integers less than $\left|C\right|$ .

The MCC values for 11 microorganisms are shown in Supplementary Table 2, and in conjunction with the microbial interaction network, the key microorganisms were *Staphylococcus hemolyticus*, *Enterobacter cloacae*, *Paracoccus mutanolyticus*, *Staphylococcus aureus*, *Pasteurella multocida*, *Burkholderia pseudomallei* and *Escherichia coli*.

In Section 3.2, we identified 74 genes for constructing the regulatory network. By combining the expression levels of 74 genes with the clinical survival data of 473 patients, we used multivariate Cox analysis to identify 20 genes for risk score calculation. Patients were classified into high-risk and low-risk groups according to a median risk score of 1.41, and the classification results are shown in Figure 5C. Survival analysis revealed significant differences in survival between the high-risk and low-risk groups (*p* < 0.001; Figure 5B). The median survival time for the low-risk group was more than 10 years, with 3-year and 5-year survival rates of approximately 90% and 80%, respectively. In contrast, the high-risk group had a median survival time of less than 5 years, with 3-year and 5-year survival rates of approximately 65% and 50%, respectively. This finding indicates a lower overall survival rate and poorer prognosis for patients in the high-risk group.

A comparison of the microbial abundance data between the high-risk and low-risk groups revealed significant differences in four microorganisms (Figure 5D), three of which also exhibited high MCC values in the microbial interaction network. *Enterobacter cloacae*, *Staphylococcus haemolyticus* and *B. pseudomallei* exhibit significant differences between high- and low-risk groups. They also play key roles in the microbial interaction network. *Enterobacter cloacae*, belonging to *Enterobacteriaceae*, is a gram-negative bacterium in the gut microbiota. It can infect the human body as an opportunistic pathogen. This pathogen shows strong antibiotic resistance and may cause postoperative complications, such as sepsis and bacterial infections (E Pages and DAVIN, 2015). Asif et al. (2021) reported that digestive tract bacteria might damage pancreatic cells, increasing the risk of malignancy. Experiments have shown that *E. cloacae* causes significant DNA damage and cell death (Asif et al., 2021). *Staphylococcus haemolyticus* is a key species of *Staphylococcus* associated with infections in hospital settings; it also exhibits strong antibiotic resistance and can cause organ infections and sepsis (Takeuchi et al., 2005). *Burkholderia pseudomallei*, a pathogenic human pathogen with intrinsic antibiotic resistance, can easily cause infections with a mortality rate of 40% or higher (Wiersinga et al., 2006).

Previous studies and our findings support that *E. cloacae*, *S. haemolyticus*, and *B. pseudomallei* are associated with patient prognosis, indicating their potential as prognostic biomarkers for CRC. Our findings regarding the role of microbes in CRC prognosis are consistent with previous studies highlighting the impact of microbiota on CRC progression (Xie et al., 2022).

## 4 Conclusion

We propose a novel algorithm named CSER to construct gene regulatory network based on causal strength and ensemble regression. CSER quantifies gene correlations and infers regulatory direction and type, i.e., activation or inhibition. CSER demonstrated high accuracy on simulated datasets and identified seven key regulatory genes influencing CRC development in real datasets. From a multiomics perspective, we conducted a comprehensive analysis of genes within the regulatory network, immune cells, and microbiome data, revealing additional interactions between the CRC gene regulatory network and both the immune microenvironment and TME. As a result, we identified 12 immune cells and 3 microorganisms associated with CRC. These findings provide new biomarkers for predicting CRC and patient prognoses. Despite the potential of the CSER algorithm, further validation in larger datasets is needed to confirm its accuracy and applicability. Additionally, clinical trials are required to assess the effectiveness and reliability of the identified biomarkers for CRC diagnosis and treatment in the future.

## Data Availability

The datasets presented in this study can be found in online repository. The name of the repository and accession numbers can be found in the Supplementary material.
