# Supplementary material for: CSER: a gene regulatory network construction method based on causal strength and ensemble regression
Source: Front Genet. 2024 Sep 20;15:1481787. doi: 10.3389/fgene.2024.1481787 (PMC11449711; doi:10.3389/fgene.2024.1481787)
Supplement: Supplementary file 1 [file Table1.docx]

Supplementary Material

# Supplementary Tables

**Supplementary Table 1.** The detailed information of simulated datasets

| **Dataset** | **Genes** | **Positive edges** | **Negative edges** | **Samples** |
| --- | --- | --- | --- | --- |
| A | 200 | 238 | 237 | 200 |
| B | 400 | 532 | 539 | 400 |
| C | 500 | 872 | 950 | 500 |

**Supplementary Table 2.** The MCC values for 11 microorganisms

| **Microbe** | **MCC** |
| --- | --- |
| *Staphylococcus haemolyticus* | 36 |
| *Enterobacter cloacae* | 36 |
| *Paracoccus mutanolyticus* | 30 |
| *Staphylococcus aureus* | 24 |
| *Pasteurella multocida* | 24 |
| *Burkholderia pseudomallei* | 12 |
| *Escherichia coli* | 7 |
| *Hydrogenophaga sp.NH-16* | 2 |
| *Halomo0s sp.JS92-SW72* | 2 |
| *Clostridium botulinum* | 2 |
| *Cutibacterium acnes* | 1 |

**Supplementary Table 3.** The features of GRN construction algorithms

| **Algorithms** | **Direction** | **Type of interactions** | **Characteristics** |
| --- | --- | --- | --- |
| GENIE3 | directed | weighted | The weights do not have statistical significance and are used only for ranking. |
| PoloBag | directed | signed; weighted | Speed and accuracy may be limited by the sample features in the dataset. |
| SIREN | undirected | signed; weighted | Inference of regulatory types must be based on a known network. |
| WGCNA | undirected | weighted | Focuses on the association between modules and traits |
| CSER | directed | signed; weighted | Improves recognition accuracy and infers both the direction and type of regulation |
